# Postnatal Outcome After Ultrasound Findings of an Abnormal Fetal Gallbladder: A Systematic Review and Meta‐Analysis

**DOI:** 10.1002/pd.6719

**Published:** 2024-12-19

**Authors:** Desislava Markova, Tsvetomira Markova, Pranav Pandya, Anna L. David

**Affiliations:** ^1^ Burjeel Hospital Fetal Medicine Unit Abu Dhabi UAE; ^2^ Fetal Medicine Unit Elizabeth Garrett Anderson Wing University College Hospital London UK; ^3^ Elizabeth Garrett Anderson Institute for Women's Health University College London London UK; ^4^ Khoula Hospital Wattayah Health Center Muscat Muscat Oman; ^5^ NIHR University College London Hospitals Biomedical Research Centre London UK

**Keywords:** abnormalities, fetal gallbladder, neonatal outcome, systematic review

## Abstract

**Objective:**

To describe postnatal outcome following the prenatal diagnosis of an abnormal fetal gallbladder.

**Methods:**

We conducted a systematic review of studies from January 1980 to January 2023 that described FGB abnormalities, which included agenesis or non‐visualisation, abnormal content presence of sludge, abnormal shape or size and abnormal position, and postnatal outcome to determine the association with pathology.

**Results:**

In 51 studies, 842 fetuses had abnormal FGB. Non‐visualisation of the FGB was the most common diagnosis (521 fetuses, mean gestational age 21.6 weeks, range 14–29). The FGB was subsequently visualised prenatally in 128 out of 521 cases (24.6%; 95% CI, 20.9%–28.3%). Of the 393 cases with persistent FGB non‐visualisation (75.4%; 95% CI, 71.7–79.1), 48 cases (12.2%; 95% CI, 9.0–15.5) underwent termination of pregnancy (TOP) with FGB agenesis confirmed in 16 out of 26 fetuses that had a postmortem examination (61.5%; 95% CI, 42.8–80.2). After excluding cases with missing outcomes (*n* = 121), postnatal ultrasound was performed in 82.4% of cases with persistent non‐visualised FGB (224/272; 95% CI, 77.8%–86.9%). The gallbladder was not visualised in 63.4% (142/224; 95% CI, 57.1–69.7), confirming GB agenesis. This was an isolated finding in 41.1% of cases (92/224; 95% CI, 34.6–47.5). Of 272 known outcomes, biliary atresia, cystic fibrosis, and structural or chromosomal abnormalities were diagnosed in 8.5% (*n* = 23), 12.5% (*n* = 34), 18.0% (*n* = 49) and 6.3% (*n* = 17) cases, respectively. The sensitivity (true positive rate) of ultrasound for GB agenesis in fetuses with persistently non‐visualised FGB was 58.1% (158/272; 95% CI, 52.2%–64.0%). Fetal gallbladder stones/sludge were described in 100 fetuses mainly in the third trimester of pregnancy (mean gestational age 33.8 weeks). Resolution of postnatally followed up cases occurred in around one‐third of the cases (37.3%) within 1 month after birth. There was a low reported association with severe conditions (2%).

**Conclusions:**

This systematic review and meta‐analysis found that when the fetal gallbladder was absent in mid‐trimester, it was visualised in subsequent fetal ultrasound examinations in around 25% of cases. If persistently absent on prenatal ultrasound, the confirmed rate of GB agenesis was around 50%, with the neonates having biliary atresia, cystic fibrosis, or structural abnormalities. Because of the association with severe conditions, if persistent FGB agenesis is suspected, prenatal diagnosis should be offered. FGB abnormalities such as stones/sludge tended to resolve by 1 year of age with around half of all cases resolving by 1 month postnatal.

**Summary**

- What's already known about this topic?
  ◦ Fetal gall bladder abnormalities although rare, are important as they can be associated with aneuploidy, biliary atresia, and single gene disorders such as cystic fibrosis.
- What does this study add?
  ◦ This systematic review found that when the fetal gallbladder was absent on mid‐trimester ultrasound, it was subsequently visualised later in pregnancy in around 25% of cases; if persistently absent, gall bladder agenesis was confirmed postnatally in 58% of cases.
  ◦ This study aids parental counseling after prenatal diagnosis of abnormal fetal gallbladder: parents can be reassured in fetal gallbladder abnormalities such as stones/sludge that they mainly resolve by 1 year of age with around half resolving by 1 month postnatally.

## 1 Introduction

Abnormalities of the fetal gallbladder (FGR) are rare with a reported incidence of about 0.15% [1]. Although examination of the FGB is not part of routine ultrasound protocols, FGB abnormalities are important as they can be associated with aneuploidy, biliary atresia, and single gene disorders such as cystic fibrosis. Based on prenatal ultrasound findings, FGB abnormalities can be characterised into four groups (fetal gall‐bladder agenesis or non‐visualisation, abnormal content (cholelithiasis or fetal gallbladder stones, presence of sludge), abnormal shape and size (duplication or enlarged FGB) and abnormally positioned FGB (left‐sided or floating FGB).

FGB agenesis has an incidence of < 1 in 6000 livebirths [2]. The reported incidence of isolated FGB non‐visualisation is higher in the second trimester, however, ranging between 1 in 300 and 1 in 9000 [3, 4]. The variability may be due to difficulties with FGB visualisation rather than true agenesis, making parental counseling difficult. Abnormal FGB content is rare (incidence 0.07%–1.15% [5]) and much less common than the 10% incidence in the adult population [1, 6]. Fetal gallstones appear as hyperechogenic structures within the gallbladder that cause posterior acoustic shadowing when stones are larger than 3 mm, independent of the calcium content. When stones are smaller than 3 mm (microlithiasis), the typical ultrasound appearance is of gallbladder ‘sand’ or ‘sludge’ or multiple echogenic foci, which due to their density usually does not cast an acoustic shadow.

FGB duplication is rare (reported incidence 1 in 3000 to 1 in 4000 [7]). Enlarged FGB (area of FGB > 2SD above the mean area for gestational age), left sided FGB (FGB on the left side of the umbilical vein, between the stomach and the umbilical vein) and ‘floating’ FGB (mobile FGB on different ultrasound examinations and usually found ‘floating’ between the bowel loops) are even less common [1].

Counseling parents when there is an abnormal FGB can be complex as data on neonatal outcomes after diagnosis is inconsistent. Our objective was to provide data on the confirmation of ultrasound‐guided prenatal diagnosis, and resolution or persistence of the findings in the neonate when a prenatal diagnosis of abnormal FGB is made.

## 2 Methods

This is a systematic review and a meta‐analysis of the published literature about abnormalities of FGB between January 1980 and January 2023. A MEDLINE and Web of Science search of journal articles was performed electronically for the above mentioned 40 years period. The search was for (fetal OR antenatal* OR prenatal*) AND gallbladder AND abnormalities* OR non‐visualisation* OR duplication* OR gallstones* OR cholelithiasis* OR enlarged* OR left‐sided AND Humans*.

Titles and abstracts were screened for relevance by two reviewers (D.M. and T.M.). All relevant articles were read in full and reviewed by D.M. and T.M. Disagreements were resolved through discussion and consensus. When more than one study was published for the same group of patients, the study with the most detailed information was included to avoid duplication.

References were managed using ENDNOTE software. The study protocol was modified according to the Preferred Reporting Items for Systematic Review and Meta‐Analysis guidelines.

The study population was any patient with a prenatal ultrasound diagnosis of abnormal FGB. The outcomes investigated were postnatal persistence or resolution of prenatal findings or confirmation of prenatal diagnosis at initial and subsequent follow‐up. Outcomes were defined as confirmed either in cases where there was data from postnatal ultrasound follow‐up imaging or where postmortem examination was performed following termination of pregnancy (TOP). Non‐human or animal studies, studies outside the study period and conference abstracts were excluded. Studies included were of English only. Retrospective, prospective, case‐series and case report full text articles were considered eligible. The systematic review and meta‐analysis were registered with PROSPERO (registration number CRD42018094431).

### 2.1 Data Management

Data for each eligible study was entered in Excel (Microsoft) spreadsheets independently and then subsequently reconfirmed. The following variables were collected for the FGB abnormalities: ultrasound findings at diagnosis, gestational age at diagnosis, gender of the fetus, presence or absence of associated abnormalities, results of invasive testing, confirmation of diagnosis at postmortem examination (if TOP performed), or persistence/resolution of the prenatal ultrasound findings at initial and/or subsequent follow up, timing at initial and/or subsequent follow up. Studies were assessed for heterogeneity and data were meta‐analysed.

### 2.2 Statistical Analysis

Between‐study heterogeneity was explored using the index *I* ^2^ that represents the percentage of between‐study variation that is due to heterogeneity rather than chance. The *I* ^2^ index does not depend on the number of studies and the type of outcome data and is used to quantify the impact of heterogeneity and assess inconsistency. *I* ^2^ is expressed as a percentage from 0% (no heterogeneity among studies) to 100% (heterogeneity among studies).

*I* ^2^ = 100% *x* (*Q*–df)/*Q*, where *Q* is distributed as a chi‐square statistic with *k* (number of studies) minus 1 degree of freedom [8]. Risk of bias assessment was performed on the included studies using the QUADAS‐2 tool [9].

## 3 Ethical Approval

Ethical approval was not required as this was a study of already published data.

## 4 Results

### 4.1 Summary of the Results

**FIGURE 1:**
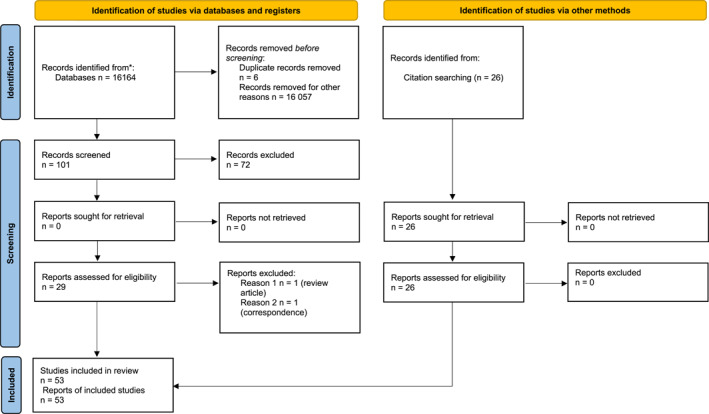
PRISMA flow diagram.

MEDLINE and Web of Science search yielded 16164 articles, the first article was published in 1983. An additional 26 records were identified through analysis of references in these identified records that were not detected in the defined search strategy. Additional searches were conducted for each specific type of FGB abnormality to identify all relevant papers for inclusion. The PRISMA flow diagram is shown in Figure 1. Of 51 included studies, there were 842 cases with prenatally diagnosed FGB abnormalities (Table 1).

**TABLE 1:** Summary of data from studies included in the systematic review.

	Non‐visualisation of FGB	Duplication of FGB	Enlarged FGB	FGB stones/sludge	Left‐sided FGB	Floating FGB
Ultrasound findings	Empty FGB fossa, non‐visualisation of FGB	Presence of two fluid filled containing structures/parallel double tubular structure	FGB dimensions > 2SD [10, 11, 12, 13]	Single or multiple or single echogenic focus, foci, mass, or material	FGB is on the left side of UV	FGB is inferior and to the left side of UV
Number of studies	16	6	4	27	1	1
Number of fetuses with prenatal diagnosis	521	8	206	100	6	1

Abbreviations: FGB, fetal gall bladder; UV, umbilical vein.

The quality assessment of the included studies is presented in Figure S1 and Table S1 and S2. The main source of bias was patient selection because the study type being mainly case series and case reports. Incomplete outcome data, selective reporting, and lack of consistency in performing initial and subsequent follow‐up postnatally resulted in other sources of bias. The analysis of the heterogeneity among the studies revealed a low risk of heterogeneity (Table S3).

In 842 cases of prenatally diagnosed FGB abnormalities, the mean gestational age at prenatal diagnosis was 23.1 (median 22.3 weeks, range 14–44 weeks). Fetal karyotype was performed in 65.6% of cases 552/842 cases (65.6%; 95% CI, 62.3–68.8). Gender was reported in 36 studies (36/51; 70.6%; 95% CI, 58.1%–83.1%). Male to female ratio, which was reported in 212 out of 624 cases, was 1.4:1.

### 4.2 FGB Non‐Visualisation

**FIGURE 2:**
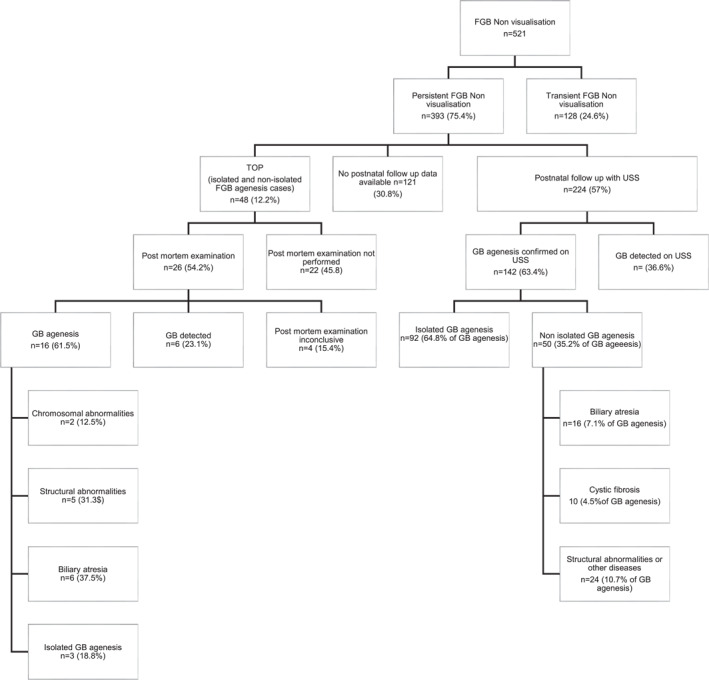
Flow chart of cases of FGB Non‐Visualisation. FGB, fetal gall bladder; GB, gall bladder; TOP, termination of pregnancy.

Sixteen studies [1, 4, 14, 15, 16, 17, 18, 19, 20, 21, 22, 23, 24, 25, 26, 27] including 521 fetuses diagnosed prenatally with FGB non‐visualisation were included (Figure 2, Table S4). The most common ultrasound description was empty FGB fossa or non‐visualisation of FGB. The average gestational age at diagnosis was 21.6 weeks (median 22 weeks, range 14–29 weeks). The male to female ratio when reported was 1.3:1 (111 cases where gender was reported out of 521 cases). Invasive prenatal diagnosis was performed in 396/521 cases (76.0%; 95% CI, 72.3–79.7). Chromosomal microarray analysis was performed in two studies and traditional karyotyping was performed in the remaining studies.

If FGB was not visualised at the ultrasound scan, a follow‐up fetal ultrasound scan was performed within a 1–3‐week period. In 24.6% (128/521; 95% CI, 20.9–28.3), the FGB was subsequently visualised prenatally (Transient FGB Non‐Visualisation, false positive). Therefore, non‐visualisation of the FGB persisted in the remaining 75.4% cases (393/521; 95% CI, 71.7–79.1). FGB was referred to as isolated if it was the only finding and non‐isolated if there was an association with either structural or chromosomal/genetic abnormalities.

#### 4.2.1 Termination of Pregnancy

Where non‐visualisation of the FGB persisted on subsequent scans or in the case of association with either structural or chromosomal/genetic abnormalities, 12.2% of cases underwent termination of pregnancy (TOP) (48/393; 95% CI, 9.0–15.5). In 26 cases of TOP (54.2%; 95% CI, 40.1–68.3), results of a post‐mortem examination were reported, with FGB agenesis confirmed in 61.5% (16/26; 95% CI, 42.8–80.2). In 15.4% of cases (4/26; 95% CI, 1.5–29.3) the post‐mortem results were inconclusive due to the destructive nature of the termination procedure. In the remaining 23.1% of cases (6/26; 23.1%; 95% CI, 6.9–39.3), FGB was detected and described as small, hypoplastic, dysplastic, or rudimentary.

#### 4.2.2 Postnatal Follow Up

In 121 (30.8%; 95% CI, 26.2–35.4) cases of persistent non‐visualised FGB, there was no evidence that postnatal ultrasound had been performed; therefore, conclusions cannot be drawn on whether the gallbladder was present or absent. After excluding cases that underwent TOP (*n* = 48) and those with missing outcomes (*n* = 121), postnatal ultrasound was performed in 82.4% of cases with persistent non‐visualised FGB (224/272, 95% CI, 77.8%–86.9%). The gallbladder was visualised in 36.6% of these imaged neonates (82/224; 95% CI, 30.3–42.9).

In those cases, with postnatal follow up after persistent non‐visualisation of FGB, GB agenesis was confirmed by ultrasound examination in 63.4% of imaged neonates (142/224; 95% CI, 57.1–69.7). GB agenesis was an isolated finding in 41.1% of imaged neonates (92/224; 95% CI, 34.6–47.5), but was found in association with biliary atresia in 7.1% (16/224; 95% CI, 3.8–10.5), cystic fibrosis in 4.5% (10/224; 95% CI, 1.8–7.2) and with prenatally diagnosed structural abnormalities in 10.7% (24/224; 95% CI, 6.7–14.8) of imaged neonates. Of all neonates diagnosed with GB agenesis, it was an isolated finding in 64.8% (92/142; 95% CI, 56.9–72.6), the remainder were associated with biliary atresia, cystic fibrosis, or other structural abnormalities.

Figure 2 illustrates the outcomes of isolated persistently absent FGB during pregnancy. In summary, the sensitivity (true positive rate) of ultrasound for GB agenesis in fetuses with persistently non‐visualised FGB (224 cases), confirmed either by postmortem examination after TOP (16 cases) or by neonatal ultrasound (142 cases), was 58.1% (158/272; 95% CI, 52.2%–64.0%). As not all studies stated the gestational age when the follow‐up ultrasound scan was performed, it is not possible to identify at which gestational age the FGB will be detected with 100% certainty.

#### 4.2.3 Association of FGB Non‐Visualisation With Underlying Anomalies

Of 272 fetuses with persistent FGB non‐visualisation and complete outcome data, 123 had an underlying anomaly (45.2%, 95th CI, 39.3%–59.1%).

Biliary atresia was diagnosed in 8.5% of cases with FGB non‐visualisation on ultrasound (23/272, 95% CI, 5.1%–11.8%).

In 30.4% (7/23; 95% CI, 11.6–49.2), TOP and subsequent post‐mortem examination confirmed GB agenesis in 6 cases (6/7; 85.7%; 95% CI, 59.8–111.6) and in one case, a hypoplastic GB was detected. In the remaining 16 cases (16/23; 69.6%; 95% CI, 50.8–88.4), GB agenesis was confirmed postnatally, and patients underwent surgery for biliary atresia (Figure S2).

Cystic fibrosis was diagnosed in 12.5% of cases with FGB non‐visualisation on ultrasound (34/272, 95% CI, 8.6%–16.4%; Figure S3). Termination of pregnancy was performed in 32.4% of cases (11/34; 95% CI, 16.6–48.1), with post‐mortem examination performed in 36.4% of terminated fetuses (4/11; 95% CI, 7.9–64.8), where the GB was always detected. GB agenesis was confirmed in all neonates with cystic fibrosis that had follow‐up (10/23; 43.5%; 95% CI, 23.2–63.7).

In all cases of biliary atresia or cystic fibrosis, there were other signs of these diseases such as sonographic gastrointestinal abnormalities (e.g., dilated or echogenic bowel), cleft lip and palate, and severe fetal growth restriction. There were also abnormal fetal digestive enzymes (gamma glytamyl transpeptidase, GGTP, or intestinal alkaline phosphatase isoenzyme, ALP) on amniotic fluid analysis provided by amniocentesis.Chromosomal abnormalities were diagnosed in 6.3% of cases with FGB non‐visualisation (17/272, 95th CI, 3.4%–9.1%). Termination of pregnancy was performed in most cases (88.2%, 15/17; 95% CI, 72.9–103.6), and post‐mortem examination in two of these TOPs in which GB agenesis was confirmed (one case with (46xxadd(14) (q32.1) and one case with DiGeorge syndrome). In the remaining two cases, there was no clear data on whether the patients had TOP or had live birth (Figure S4). Trisomy 18, trisomy of the short arm of chromosome 9, triploidy, triple *X*, balanced translocation (5q, 16p) and P/LP CNV were the other abnormalities detected in the TOP group.

Associated structural abnormalities were detected in 18.0% of fetuses with FGB Non‐Visualisation (49/272; 95% CI, 13.4%–22.6%). Termination of pregnancy was performed in 20.4% (10/49; 95% CI, 9.1–31.7). Postmortem examination was performed in five cases which confirmed GB agenesis in the fetus. The following structural abnormalities were observed—club feet, cystic hygroma, micrognathia, absence of radius and hand, mesomelia, adactyly, hydrops fetalis) (Figure S5). GB agenesis was confirmed in 61.5% (24/39; 95% CI, 46.3–76.8) neonates with prenatally diagnosed structural abnormalities.

#### 4.2.4 Timing of Neonatal Follow Up

Most studies (9/16; 56.3%) did not specify the time of postnatal ultrasound examination, reporting only that the examination was performed after birth in the postnatal period. The earliest neonatal follow‐up was on day one after birth and the latest on day 16 (median 11 days, mean 9.3 days, range 1–16). The systematic review found that a second neonatal follow‐up was performed in 3.1% of live births with persistent GB non‐visualisation that had an initial neonatal US follow up (*n* = 7/224; 95% CI, 0.8–5.4). In all 7 cases, the diagnosis of GB agenesis was reconfirmed. The average timing of the second neonatal follow‐up was 6.5 months (median‐6.5 months, range 1–12 months).

### 4.3 Abnormal Content of FGB (FGB Stones, Presence of Sludge)

**FIGURE 3:**
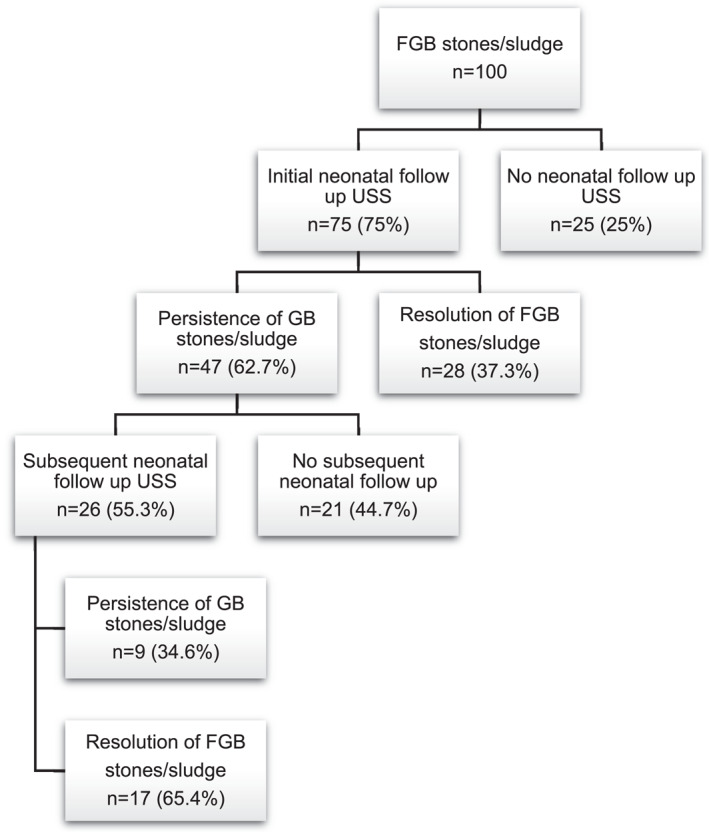
Neonatal outcomes for FGB stones/sludge.

Twenty‐seven studies [5, 28, 29, 30, 31, 32, 33, 34, 35, 36, 37, 38, 39, 40, 41, 42, 43, 44, 45, 46, 47, 48, 49, 50, 51, 52, 53] describing 100 fetuses diagnosed prenatally with FGB stones/sludge were included (Figure 3, Table S5). A single or multiple echogenic focus/focus in the FGB with or without acoustic shadowing was commonly described. The shadowing was seen either as posterior or distal shadowing or as a comet‐tail or a v‐tailed shadowing artifact. Sludge was presented either as diffuse or echogenic material filling FGB. The mean gestational age at diagnosis was 33.8 weeks (median 35 weeks, range of 19–44 weeks). The male to female ratio when reported was 1.5:1 (92/100 cases).

Invasive prenatal diagnosis was performed only in 7% cases (7/100; 95% CI, 2%–12%), for associated ultrasound findings ‐ structural abnormalities (non‐isolated abnormal FGB content cases, *n* = 3, atrioventricular septal defect, Tetralogy of Fallot, club foot, neck oedema), for twin‐to‐twin transfusion syndrome in a monochorionic‐diamniotic twin pregnancy (*n* = 1), two cases due to polyhydramnios and in one case for lung maturity. Aneuploidy was detected in 2% of cases (2/100, 1 case trisomy 21, 1 case of chromosomal rearrangement/translocation (46, XX, *t*(10, 11) (q22; q22)). Karyotype was normal in the remaining 4% of tested cases. There were no cases of TOP reported. In all these cases that were karyotyped, the abnormal content of FGB was diagnosed at a later gestational age than the decision for invasive testing.

In one‐third of the studies (8/27 studies; 16/100 cases; 16%; 95% CI, 8.8%–23.2%) when FGB stones/sludge was suspected at an earlier gestational age, patients were invited to attend a follow‐up ultrasound scan to monitor the evolution of the findings during pregnancy. Gestational age at follow up fetal ultrasound was reported in 75% of studies performing follow up (6/8, mean GA 35.7, median 36.2, range 30–40 weeks; 6 cases). Resolution in pregnancy occurred in 3 cases (3/16; 18.8%).

Neonatal sonographic follow‐up was performed as early as the first postnatal day (mean 17.2, median 4, range 1–84 days). Three quarters of neonates were followed up postnatally (75/100; 95% CI, 66.5%–83.5%). In 62.7% of the neonates examined (47/75; 95% CI, 51.7%–73.6%), the prenatal ultrasound finding of FGB stones/sludge persisted, giving resolution at the first neonatal follow up in 37.3% of cases (28/75; 95% CI, 26.4%–48.3%). The remaining 55.3% of cases of persistent postnatal GB stones/sludge underwent a second ultrasound examination (26/47 cases, 95% CI, 41.1%–69.5%), of which only 34.6% (9/26; 95% CI, 16.3%–52.9%) had continuing evidence of cholelithiasis, with resolution seen in 65.4% cases (17/26, 95% CI, 47.1%–83.7%). The mean timing of subsequent neonatal follow‐up was 34.3 weeks postnatal (median 12, range 1–216 weeks). GB stones/sludge were confirmed to resolve on the initial postnatal USS (37.3%, 28/75 cases) or subsequent postnatal USS (65.4%, 17/26 cases).

### 4.4 FGB Duplication

We identified six studies [1, 54, 55, 56, 57, 58] including eight fetuses diagnosed prenatally with FGB duplication, most commonly described as two fluid‐filled containing structures or parallel double tubular structures (Table S6). Eighty‐eight percent of cases were isolated (7/8, 95% CI, 64.6%–110.4%), and one case was associated with structural abnormalities/left sided gallbladder and bilateral renal agenesis (non‐isolated FGB duplication)/. The mean gestational age at diagnosis was 25 weeks (median 25, range 20–32 weeks. Male to female ratio when reported was 2:3 (5 cases).

Invasive prenatal testing (amniocentesis or cordocentesis) for karyotyping was performed in 25% of cases (2/8, 95% CI, 0.5%–53%), with one chromosomal abnormality identified that was subsequently found to be carried by the mother (46, XX, *t*(X; 10) (p11.2; q24.3) [58]) who was also found to have a duplicate gallbladder. The other case underwent TOP due to renal agenesis, with duplicate FGB confirmed on post‐mortem examination.

Six neonates underwent follow up in which duplicate GB was confirmed by neonatal ultrasound in all 6 cases (100%). A second neonatal scan in two cases (day 4 and 1 month postnatal) reconfirmed the diagnosis.

### 4.5 Enlarged FGB

Four studies [10, 27, 55, 59, 60] including 206 fetuses diagnosed prenatally with enlarged FGB were included in the review (Table S7). The most common ultrasound description was FGB area > 2SD for the corresponding gestational age. Mean GA of diagnosis when reported was 23.7 weeks (median 24 weeks). Fetal gender was not reported. Amniocentesis was performed in 74.3% (153/206, 95% CI, 68.3%–80.2%) with an abnormal result in 3.9% (8/206, 95% CI, 1.2%–6.5%): Trisomy 18 in 1% (2/206, 95% CI, −0.4%–2.3%) and in 2.9% Pathogenic/Likely Pathogenic Copy Number Variants were detected (6/206, 95% CI, 0.6%–5.2%) that all subsequently underwent TOP. In a further two cases with enlarged FGB and concomitant multiple abnormalities (non‐isolated enlarged FGB), fetal karyotyping was declined and trisomy 13 and 18 were diagnosed postnatally. The overall rate of chromosomal abnormalities was therefore 4.9% (10/206, 95% CI, 1.9%–7.8%). After excluding the two neonates with aneuploidy, neonatal ultrasound assessment was reported in only 16 cases; timing of the assessment was not described but, in all cases, GB dimensions were within the normal limits.

### 4.6 Left‐Sided FGB

One study was identified that reported six fetuses diagnosed with left‐sided FGB was included, where the FGB was positioned on the left side of the intrahepatic umbilical vein [1]. There were no other signs of heterotaxy reported. Mean gestational age of diagnosis was 19 weeks (median 18, range 15–24 weeks). The reported male to female ratio was 1:2 (*n* = 6). Invasive prenatal testing for conventional karyotyping was performed in 3 cases (50%) with normal results. Due to additional fetal abnormalities (renal agenesis syndrome and hypoplastic left heart) in two cases (non‐isolated left‐sided FGB), the pregnancies were terminated (2/6; 33%), with left‐sided FGB confirmed on post‐mortem examination. In the remaining four cases, subsequent fetal serial ultrasound scans confirmed the malposition of FGB. No neonatal ultrasound follow‐up was reported.

### 4.7 ‘Floating’ FGB

Only one study including one fetus diagnosed prenatally, at 15 weeks of gestation with ‘floating’ FGB, was included in the review (Table 1). ‘Floating’ FGB may be completely invested by the peritoneum with no mesentery, may be suspended from the liver by a complete mesentery, or the neck of FGB may have a mesentery with a cystic artery in it, while the fundus and the body are free. ‘Floating’ FGB was found to the left of the midline on the left side of the intrahepatic umbilical vein. Unlike left sided FGB, it did not have a steady location adjacent to the stomach but was ‘floating’ between the hyperechogenic bowel loops. The location of FGB was not constant and was different from several examinations [1]. The fetus was female with a normal karyotype. The outcome of pregnancy was late miscarriage at 18 weeks with no post‐mortem examination; hence, there was no confirmation of the prenatally diagnosed FGB abnormality.

## 5 Discussion

In this systematic review and meta‐analysis, we assessed ultrasound findings and postnatal outcomes of FGB abnormalities, focusing on neonatal persistence of gallbladder abnormalities to determine the true association with pathology. To ensure accurate data for patient counseling, we focused on confirmation of FGB abnormalities either on post‐mortem examination after TOP or on postnatal neonatal follow‐up ultrasound.

FGB non‐visualisation was mainly a second trimester diagnosis. When the fetal gallbladder was absent in mid‐trimester, it was visualised in subsequent fetal ultrasound examinations in one‐quarter of cases. If persistently absent on prenatal ultrasound, the confirmed rate of gallbladder agenesis was 63.4%. There are no national or international guidelines that recommend visualisation of the FGB for anomaly screening; nevertheless, non‐visualised FGB may occur with severe disease and is important to detect Around 35.2% of fetuses with persistently non‐visualised GB had an underlying anomaly, including structural or chromosomal anomalies, biliary atresia, or cystic fibrosis. It is important to note that of the fetuses that had persistent non‐visualisation of FGB, 12% underwent termination of pregnancy. Of the half that had a post‐mortem examination, 23% of fetuses had a detectable gallbladder and a further 15% of examinations were inconclusive. This highlights the risk that ultrasound is not always able to detect the FGB, and other imaging modalities such as Magnetic Resonance Imaging may be a useful adjunct when the FGB is not visualised on ultrasound.

A previous systematic review of FGB non‐visualisation calculated different rates of isolated FGB agenesis, biliary atresia, cystic fibrosis and chromosomal abnormalities [23]. However, our search identified 16 studies compared to the seven in this review [23]. In addition, we only included cases of non‐visualisation that were persistent and confirmed either on fetal post‐mortem examination or neonatal ultrasound. Our results indicate that the inability to visualise the FGB should prompt clinicians to perform a detailed fetal anomaly scan to detect other abnormalities. Because of the association with severe conditions, if persistent FGB agenesis is suspected, invasive testing should be offered to rule out chromosomal abnormalities, cystic fibrosis, or biliary atresia, with testing for digestive enzymes in the amniotic fluid [14]. Most included studies did not report when neonatal ultrasound follow‐up was performed and in those studies that did, timing of ultrasound was variable. A repeat neonatal scan was performed in only seven cases, with the diagnosis reconfirmed. Our findings suggest that the initial neonatal ultrasound can be performed within a few days of birth in cases of non‐visualised FGB.

FGB stones/sludge was mainly a third trimester diagnosis, explaining why two‐thirds of studies did not report a subsequent fetal ultrasound scan. Most neonates were offered at least one postnatal ultrasound examination, when in 37.3% of cases, the abnormal contents had resolved. Subsequent neonatal ultrasound examination was performed in just over half of the cases, with the abnormality resolved in two‐thirds. Therefore, it can be concluded that nearly half of the abnormal gallbladder contents identified during fetal life resolved postnatally (45%). Oral feeding and postnatal hydration are thought to stimulate the resolution or spontaneous passage of stones/sludge through the biliary tree [34]. Although Brown et al. suggested that the resolution was more likely to occur if the echogenic material does not cause distal shadowing [34], we were unable to confirm this finding due to insufficient information provided in the included studies.

FGB stones/sludge were generally not associated with chromosomal abnormalities or genetic disorders, and invasive prenatal diagnosis was performed in only 7% of cases. For optimal counseling, it is best to inform patients/couples of the chance of there being aneuploidy detected against the risk to the pregnancy of amniocentesis so that they can then make an informed decision that best fits their personal circumstances. We found a low reported association with severe conditions; thus, parents can be reassured that most babies with FGB stones/sludge will have a good outcome. Abnormal production, secretion and transportation of bile have been suggested for the formation of FGB stones or sludge [28]. Because of insufficient data from the included studies, we are unable to identify risk factors that may predispose to the formation of abnormal FGB content. Future studies are recommended to comprehensively report the number of echogenic focus/foci, size of the echogenic foci (if ≥ 3 mm), presence of posterior acoustic shadowing and/or presence of sludge.

Duplicated FGB was diagnosed at the fetal anomaly scan in half of the cases, with the remainder receiving a third trimester diagnosis. There was a low risk of chromosomal or other associated abnormalities and patients should be reassured of a good neonatal outcome. Diagnosis may be of clinical importance in adulthood as duplicate FGB is associated with a high prevalence of cholelithiasis and intermittent cystic duct obstruction [41]. Our findings suggest that the optimum time for neonatal follow‐up ultrasound is in the early neonatal period, when the diagnosis was confirmed in all cases.

Enlarged FGB was diagnosed when the FGB was > 2SD mean area for gestational age in four studies [11, 12, 13, 61] and one study used their own normal FGB comparison group [10]. There is a need for a comprehensive normative dataset for dimensions of FGB for each gestational age. Parents can be reassured that the neonatal outcome is likely to be good, with a low rate of chromosomal abnormalities and normal gallbladder measurements detected in neonates offered postnatal ultrasound scans.

Left‐sided FGB was an uncommon diagnosis. In two out of the six cases, there were other associated fetal abnormalities confirmed on post‐mortem examination. Karyotype was not performed and therefore, we cannot conclude whether left‐sided FGB is associated with chromosomal abnormalities or genetic syndromes. In the other four cases, left sided FGB could not be confirmed postnatally due to the obliteration of the intrahepatic umbilical vein after birth. There was only one case describing ‘floating’ FGB, where the FGB was situated between the bowel and inferior and on the left side of the umbilical vein. The ultrasound appearance of ‘floating’ FGB may overlap with left‐sided FGB and therefore perhaps should not be described as a separate entity.

### 5.1 Strengths and Weaknesses

Our search criteria were wide and included studies describing confirmation of the abnormalities either after birth in neonates or at post mortem examination, allowing us to present data on resolution of abnormal FGB findings, which is useful for parental counseling. Our findings were however limited by the case descriptions in examined studies; over 95% of included studies were case reports or small case series with high risk of bias, and most were retrospective with only two prospective studies. Bias is also introduced as most cases were referrals to specialised centers. For the less common FGB abnormalities such as left sided or floating FGB, there were only a small number of cases described, making drawing conclusions difficult. There will likely be many complex cases of absent FGB that will be undetected in fetuses with multiple fetal abnormalities. The description of the ultrasound examinations varied, especially for neonatal follow‐up. Additional parameters such as maternal age, ethnicity, parity, and co‐morbidities were unavailable, and their association with FGB abnormalities was undetermined. Due to incomplete outcome reporting, the number of cases with prenatally detected FGB abnormalities with complete postnatal follow‐up data was limited. Finally, visualization of the gall bladder is not routinely screened and does not appear in any international guidelines; therefore, this study is limited by the lack of routine screening in the population.

## 6 Conclusions

Non‐visualisation and stones/sludge are the most common FGB abnormalities, with non‐visualisation/GB agenesis being most associated with pathology. Where the fetal gallbladder was absent in mid‐trimester, it was visualised in subsequent fetal ultrasound examinations in around 25% of cases. If persistently absent on prenatal ultrasound, the confirmed rate of GB agenesis was 58%, with the neonates having biliary atresia, cystic fibrosis, or structural abnormalities. Because of the association with severe conditions, if persistent FGB agenesis is suspected, prenatal diagnosis should be offered. FGB abnormalities such as stones/sludge tended to resolve by 1 year of age with around half of all cases resolving by 1 month postnatal. Abnormally shaped FGB such as duplication was confirmed after birth, and neonates with a diagnosis of enlarged FGB had a normal GB appearance at postnatal follow up. The optimum time for neonatal follow‐up depends on the type of specific FGB abnormality.

## Ethics Statement

No ethical approval was required for this systematic review.

## Consent

The authors have nothing to report.

## Conflicts of Interest

The authors declare no conflicts of interest.

## Previous Presentation

This work was presented as an abstract at the ISUOG (International Society of Ultrasound in Obstetrics and Gynecology) conference October 2020. Markova D, Markova T, Pandya P, David AL. Postnatal outcome after ultrasound findings of an abnormal fetal gall bladder: a systematic review and meta‐analysis. Abstract published in *Ultrasound in Obstetrics and Gynecology* 2020; 56(S1):81.

## Supporting information

Figure S1

Figure S2

Figure S3

Figure S4

Figure S5

Table S1

Table S2

Table S3

Table S4

Table S5

Table S6

Table S7

## Data Availability

Data sharing is not applicable to this article as no new data were created or analyzed in this study.
