# Supplementary material for: Postnatal Outcome After Ultrasound Findings of an Abnormal Fetal Gallbladder: A Systematic Review and Meta‐Analysis
Source: Prenat Diagn. 2024 Dec 19;45(2):185–95. doi: 10.1002/pd.6719 (PMC11790525; doi:10.1002/pd.6719)
Supplement: Supplementary file 1 — Figure S1 [file PD-45-185-s005.docx]

**Supplementary Figure 1. Top chart: Proportion of studies with low, high, or unclear risk of bias. Bottom chart: Proportion of studies with low, high, or unclear concerns regarding applicability.**
