# Supplementary material for: Postnatal Outcome After Ultrasound Findings of an Abnormal Fetal Gallbladder: A Systematic Review and Meta‐Analysis
Source: Prenat Diagn. 2024 Dec 19;45(2):185–95. doi: 10.1002/pd.6719 (PMC11790525; doi:10.1002/pd.6719)
Supplement: Supplementary file 2 — Figure S2 [file PD-45-185-s004.docx]

**Supplementary Figure 2. Association of FGB Non-Visualisation with biliary atresia.** TOP: termination of pregnancy, ultrasound abnormalities detected - sonographic gastrointestinal abnormalities (for example dilated or echogenic bowel), cleft lip and palate, and severe fetal growth restriction. There were also abnormal fetal digestive enzymes (gamma glytamyl transpeptidase, GGTP, or intestinal alkaline phosphatase isoenzyme, ALP) on amniotic fluid analysis provided by amniocentesis.
