# Supplementary material for: Postnatal Outcome After Ultrasound Findings of an Abnormal Fetal Gallbladder: A Systematic Review and Meta‐Analysis
Source: Prenat Diagn. 2024 Dec 19;45(2):185–95. doi: 10.1002/pd.6719 (PMC11790525; doi:10.1002/pd.6719)
Supplement: Supplementary file 5 — Figure S5 [file PD-45-185-s001.docx]

**Supplementary Figure 5. Association of FGB Non-Visualisation with structural abnormalities.** TOP: termination of pregnancy; PM: postmortem examination
