# Supplementary material for: Postnatal Outcome After Ultrasound Findings of an Abnormal Fetal Gallbladder: A Systematic Review and Meta‐Analysis
Source: Prenat Diagn. 2024 Dec 19;45(2):185–95. doi: 10.1002/pd.6719 (PMC11790525; doi:10.1002/pd.6719)
Supplement: Supplementary file 7 — Table S2 [file PD-45-185-s007.docx]

|  | **PATIENT SELECTION** | **INDEX TEST** | **REFERENCE STANDARD** |
| --- | --- | --- | --- |
| Stringer, 1995 | Low | Low | Low |
| Kesrouani, 2018 | Low | Low | Low |
| Suma, 1998 | Low | Low | Low |
| Nishi, 1997 | Low | Low | Low |
| Devonald, 1992 | Low | Low | Low |
| Kiserud, 2018 | Low | Low | Low |
| Kingensmith, 1988 | Low | Low | Low |
| Basu, 2015 | Low | Low | Low |
| Brown, 1993 | Low | Low | Unclear |
| Tam, 2010 | Low | Low | Low |
| Hertzberg, 1998 | Low | Low | Low |
| Humi, 2017 | Low | Low | Low |
| Sepulveda, 1995 | Low | Low | Low |
| Sheiner, 2006 | Low | Low | Low |
| Abbitt, 1990 | Low | Low | Low |
| Petrikovsky, 1996 | Low | Low | Low |
| Bronshtein, 1993 | Low | Low | Low |
| Maggi, 2017 | Low | Low | Low |
| Gerscovich, 2011 | Low | Low | Low |
| Kinoshita, 2002 | Low | Low | Low |
| Sifakis, 2007 | Low | Low | Low |
| Bronshtein, 1993 | Low | Low | Low |
| Bronshtein, 1993 | Low | Low | Low |
| Boughanim, 2008 | Low | Low | Low |
| Bronshtein, 1993 | Low | Low | Low |
| Shen, 2011 | Low | Low | Low |
| Ochshorn, 2007 | Low | Low | Low |
| Lena, 2018 | Low | Low | Low |
| Muller, 2015 | Low | Low | Low |
| Dreux, 2012 | Low | Low | Low |
| Hertzberg,1996 | Low | Low | Low |
| Ben-ami, 2002 | Low | Low | Low |
| Agnifili, 1999 | Low | Low | Low |
| Beretsky, 1983 | Low | Low | Low |
| Clarke, 1994 | Low | Low | Low |
| Holloway, 2010 | Low | Low | Low |
| Lariviere, 2006 | Low | Low | Low |
| Munjulury, 2005 | Low | Low | Low |
| Sepulveda,2018 | Low | Low | Low |
| Suchet, 1993 | Low | Low | Low |
| Triunfo, 2013 | Low | Low | Low |
| Troyano-Luque, 2014 | Low | Low | Low |
| Darouich, 2019 | Low | Low | Low |
| Bardin, 2016 | Low | Low | Low |
| Bergougnoux, 2019 | Low | Low | Low |
| Blazer, 2002 | Low | Low | Low |
| Dugueperoux, 2012 | Low | Low | Low |
| Pasquo, 2019 | Low | Low | Low |
| Ruiz, 2017 | Low | Low | Low |
| Comert, 2019 | Low | Low | Low |
| Sepulveda, 1995 | Low | Low | Low |
| Petrikovsky, 1995 | Low | Low | Low |
| Hertzberg, 1998 | Low | Low | Low |

**Supplementary Table 2. A comprehensive assessment of the studies using QADAS-2 tool-assessment of applicability.**
