# Supplementary material for: Postnatal Outcome After Ultrasound Findings of an Abnormal Fetal Gallbladder: A Systematic Review and Meta‐Analysis
Source: Prenat Diagn. 2024 Dec 19;45(2):185–95. doi: 10.1002/pd.6719 (PMC11790525; doi:10.1002/pd.6719)
Supplement: Supplementary file 8 — Table S3 [file PD-45-185-s012.docx]

| **n** | **Study** | ***I² -* heterogeneity index** |
| --- | --- | --- |
| 1. | Non-visualisation of FGB | 0% |
| 2. | FGB stones/sludge | 0% |
| 3. | Duplication of FGB | 0% |
| 4. | Enlarged FGB | 24% |

**Supplementary Table 3. Heterogeneity amongst studies.**
