# Supplementary material for: Postnatal Outcome After Ultrasound Findings of an Abnormal Fetal Gallbladder: A Systematic Review and Meta‐Analysis
Source: Prenat Diagn. 2024 Dec 19;45(2):185–95. doi: 10.1002/pd.6719 (PMC11790525; doi:10.1002/pd.6719)
Supplement: Supplementary file 9 — Table S4 [file PD-45-185-s010.docx]

| Study | Cases seen prenatally (n) | Cases followed up PN (n) | Finding confirmed at 1st PN US follow up  (n) | GA (mean* or range as stated) | m / f | Invasive testing (n) | Transient FGB seen.  (n) | Transient FGB absent PN (n) | TOP (n) | Reason for TOP | PM  (n) | Absent FGB confirmed at TOP (n) | Cases NOT reported on PM  (n) | Absent GB PN (n) | CF with absent FGB-(n) | BA (n) | BA with absent GB  (n) | Isolated FGB Agenesis PN  (n) |
| --- | --- | --- | --- | --- | --- | --- | --- | --- | --- | --- | --- | --- | --- | --- | --- | --- | --- | --- |
| Bardin, 2016^13^ | 32 | 12 | 5 | 18.5 | NS | 32 | 15 | 7 | 5 | 3 BA, 1 Chr abn, 1 abnormal hepatic vasc | 4 | 4 | 2 | 5 | 0 | 3 | 3 | 5 |
| Ben-ami,2002^14^ | 1 | 0 | 0 | 15 | m | 1 | N | N | 1 | 1 BA | 1 | 1 | 0 | 0 | 0 | 1 | 1 | 0 |
| Bergougnoux, 2019^15^ | 15 | 0 | 0 | 24-31 | NS | 4 | 3 | NS | 2 | 2 CF | 0 | NS | NS | NS | NS | NS | NS | NS |
| Boughanim, 2008^16^ | 4 | 3 | 1 | 22, 25 | m 2; f 1 | 3 | N | 2 | 1 | 1 CF | 0 | NS | NS | 1 | 0 | 0 | 0 | 1 |
| Blazer, 2002^17^ | 34 | 7 | 5 | 14-16 | NS | 34 | 15 | 2 | 9 | 4 triploidy, 2 CF, 3 str abn | 9 | 3 | 7 | 5 | 0 | 0 | 0 | 5 |
| Bronshtein 1993^1^ | 7 | 2 | 2 | 14,15, 29 | f 4;  m 3 | 7 | N | N | 5 | 2 for str, 3 for NVFGB | 5 | 5 | 0 | 2 |  | 0 | 0 | 2 |
| Dreux, 2012^18^ | 102 | 83 | 40 | 23* | NS | 102 | 17 | 43 | 2 | 1 chr abn, 1 BA | 2 | 2 | 0 | 22 | 10 | 8 | 7 | 22 |
| Dugueperoux, 2012^19^ | 37 | 15 | 9 | 22* | NS | 37 | 4 | 6 | 7 | 5 CF; 2 chr abn | 1 | no | PM not done in 6 cases | 9 | 0 | 0 | 0 | 9 |
| Hertzberg, 1996^20^ | 101 | / | / | 21.3* | m 49;  f 31 | 0 | 59 | 0 | 0 | / | / | / | / | / | / | / | / | / |
| Muller, 2015^21^ | 2 | 1 | 0 | 18+1; 23 | 2 f | 1 | N | 1 | 1 | 1 BA | 1 | 1 |  | 0 | 0 | 1 | 0 | 0 |
| Ochshorn, 2007^4^ | 22 | 15 | 2 | 15,16 | 9 f;  7 m | 17 | 1 | 13 | 2 | 1 CF, 1 triploidy | 2 | 0 | 0 | 2 | 0 | 0 | 0 | 2 |
| Pasquo, 2019^22^ | 16 | 8 | 4 | 23-33 | NS | 16 | 8 | 4 | 0 | 0 | / | / | / | 4 | n | 2 | 2 | 1 |
| Ruiz, 2017^23^ | 2 | 1 | 1 | 24.5,  22.8 | m 1; f 1 | 1 | 0 | 0 | 1 | 1 BA | 1 | 0 | 0 | 1 | 0 | 1 | 0 | 1 |
| Sagi-Dain, 2018^24^ | 51 | 0 | NS | 19.8* | NS | 51 | NS | NS | NS | NS | NS | NS | NS | NS | NS | NS | NS | NS |
| Shen, 2011^25^ | 21 | 10 | 6 | 16.1* | NS | 16 | 6 | 4 | 5 | 1 CF,3 chr, 1 str abn | 0 | NS | PM not done in TOP cases | 6 | / | 0 | 0 | 6 |
| Qian Y, 2022 | 74 | NS | NS | 26+4* | NS | 74 | NS | NS | 7 | Trisomy 18, trisomy 9 | NS | NS | NS | 67 | NS | NS | 7 | 22 |

**Supplementary Table 4. Summary of data for studies reporting about FGB Non-visualisation.** PN: postnatally; GA: mean gestational age or range as stated; PM: postmortem examination; NS: not stated; TOP: termination of pregnancy; BA: biliary atresia; CF: cystic fibrosis; n: number of patients; m: male; f: female; Y: yes; N: no.
