# Supplementary material for: Postnatal Outcome After Ultrasound Findings of an Abnormal Fetal Gallbladder: A Systematic Review and Meta‐Analysis
Source: Prenat Diagn. 2024 Dec 19;45(2):185–95. doi: 10.1002/pd.6719 (PMC11790525; doi:10.1002/pd.6719)
Supplement: Supplementary file 10 — Table S5 [file PD-45-185-s009.docx]

| Study | Cases seen prenatally.  (n) | Cases followed up PN.  (n) | Persistence of finding at 1^st^ PN  follow up.  (n) | Persistence of finding at 2^nd^ PN follow up.  (n) | GA (mean* or range as stated) | m/f | Invasive testing  Y/N | Karyotype  results | Associated abnormalities.  Y/N  Type | TOP  Y/N |
| --- | --- | --- | --- | --- | --- | --- | --- | --- | --- | --- |
| Abbitt, 1990^26^ | 1 | 1 | 1 | 0 | 37 | m | N | / | N | N |
| Agnifili, 1999^27^ | 3 | 2 | 2 | 0 | 20-21 | m 1.  f 2 | N | / | Y  dilated renal pelvises in 1 | N |
| Basu, 2015^28^ | 1 | 1 | 1 | 1 | 39 | f | N | / | N | N |
| Beretsky, 1983^29^ | 1 | 1 | 1 | 0 | 36 | NS | N | / | N | N |
| Brown, 1992^30^ | 26 | 15 | 10 | 3 | 36.2* | m 11.  f 15 | N | / | Y  hemivertebrae and unilateral hydronephrosis | N |
| Clarke, 1994^31^ | 1 | 1 | 1 | 0 | 19 | m | N | / | N | N |
| Devonald, 1992^32^ | 7 | 6 | 3 | 1 | 39.8* | m 5.  f 2 | Y in 1 | for lung maturity | N | N |
| Hertzberg, 1998^33^ | 2 | 2 | 1 | 0 | 32.8* | m 1.  f 1 | N | / | N | N |
| Holloway, 2010^34^ | 1 | 1 | 1 | NS | 36 | m | N | / | N | N |
| Hurni,2017^35^ | 2 | 2 | 1 | 0 | 33* | m | N | / | N | N |
| Kesrouani, 2018^5^ | 3 | 1 | 1 | NS | 25* | m 2.  f 1 | N | / | N | N |
| Kiserud, 1997^36^ | 6 | 6 | 2 | 1 | 35* | m 4.  f 2 | Y in 3 | normal  translocation, trisomy 21 | Y  Club feet  Gastroschisis  Cardiac defects | N |
| Klingensmith,1988^37^ | 1 | 1 | 1 | 0 | 24 | m | N | / | N | N |
| Lariviere, 2006^38^ | 1 | 0 | NS | NS | 35+2 | f | N | / | N | N |
| Munjulury, 2005^39^ | 2 | 2 | 2 | 0 | 34* | m 1.  f 1 | N | / | N | N |
| Nishi, 1997^40^ | 1 | 1 | 1 | 0 | 36 | / | N | / | N | N |
| Petrikovsky, 1996^41^ | 5 | 4 | 0 | 1 | 31.2* | / | N | / | Y | N |
| Sepulveda, 1996^42^ | 1 | 1 | 1 | NS | 36 | m | Y | normal | N | N |
| Sepulveda, 2018^43^ | 19 | 12 | 9 | 0 | 35.6* | m 10.  f 9 | N | / | Y | N |
| Sheiner, 2006^44^ | 4 | 4 | 0 | NS | 29-32 | m | N | / | N | N |
| Stringer, 1996^45^ | 3 | 3 | 2 | 1 | 33* | m | N | / | Y | N |
| Suchet, 1993^46^ | 1 | 1 | 1 | 0 | 36 | / | N | / | N | N |
| Suma, 1998^47^ | 2 | 2 | 2 | 0 | 33.5* | m | N | / | N | N |
| Tam Iroh, 2010^48^ | 2 | 2 | 1 | 0 | 35* | m | N | / | N | N |
| Triunfo, 2013^49^ | 1 | 1 | 1 | 0 | 35 | f | N | / | N | N |
| Troyano-Luque, 2014^50^ | 2 | 2 | 1 | 1 | 23* | m 1;  f 1 | Y in 2 | normal in 2 | N | N |
| Darouich, 2019^51^ | 1 | 0 | 0 | 0 | 37 | m 1 | N | / | Y  Fetal hydrops  Cardiomegaly  Enlarged liver | IUD |

**Supplementary Table 5. Summary of data for studies reporting about FGB stones/sludge.**

PN: postnatally; GA: mean* gestational age or range as stated (weeks); PM: postmortem examination; TOP: termination of pregnancy; NS: not stated; IUD – intrauterine death; n: number of patients; m: male; f: female; Y: yes; N: no.
