# Supplementary material for: Postnatal Outcome After Ultrasound Findings of an Abnormal Fetal Gallbladder: A Systematic Review and Meta‐Analysis
Source: Prenat Diagn. 2024 Dec 19;45(2):185–95. doi: 10.1002/pd.6719 (PMC11790525; doi:10.1002/pd.6719)
Supplement: Supplementary file 11 — Table S6 [file PD-45-185-s008.docx]

| Study | Cases seen prenatally  (n) | Cases followed up PN  (n) | Finding confirmed at 1^st^ PN  follow up  (n) | GA  (weeks) | m/f  (n) | Invasive testing  Y/N | Abnormal results  Y/N | TOP  Y/N | Associated abnormalities  Y/N  Type |
| --- | --- | --- | --- | --- | --- | --- | --- | --- | --- |
| 1. Bronshtein,1993^1^ | 2 | 1 | 1 | 20* | m 2 | Y | N | Y | Y |
| 2. Gerscovich, 2011^11^ | 1 | 1 | 1 | 20 | m | N | / | N | N |
| 3. Kinoshita, 2002^10^ | 2 | 1 | 1 | 31* | f | N | / | N | N |
| 4. Maggi, 2018^9^ | 1 | 1 | 1 | 21 | / | N | / | N | N |
| 5. Sifakis, 2007^52^ | 1 | 1 | 1 | 32 | m | N | / | N | N |
| 6. Comert, 2019^53^ | 1 | 1 | 1 | 26 | f | Y | Y | N | N |

**Supplementary Table 6. Summary of data for studies reporting about duplicate FGB.** PN: postnatally; GA: mean* gestational age or range as stated (weeks); TOP: termination of pregnancy; NS: not stated; n: number of patients; m: male; f: female; Y: yes; N: no.
