# Supplementary material for: Postnatal Outcome After Ultrasound Findings of an Abnormal Fetal Gallbladder: A Systematic Review and Meta‐Analysis
Source: Prenat Diagn. 2024 Dec 19;45(2):185–95. doi: 10.1002/pd.6719 (PMC11790525; doi:10.1002/pd.6719)
Supplement: Supplementary file 12 — Table S7 [file PD-45-185-s003.docx]

| Study | Cases seen prenatally  (n) | Cases followed up PN  (n) | Finding confirmed at 1^st^ PN  follow up  (n) | GA (weeks) | m/f  (n) | Invasive testing  Y/N  (n) | Abnormal results  Y/N  (n) | TOP  Y/N  (n) | Associated abnormalities.  Y/N  Type |
| --- | --- | --- | --- | --- | --- | --- | --- | --- | --- |
| 1. Sepulveda, 1995^54^ | 8 | 5 | 0 | 24.6* | NS | Y | Y 1 | Y 1 | Y |
| 2. Petrikovsky,1995^55^ | 11 | 11 | 0 | NS | NS | N | / | N | N |
| 3. Hertzberg, 1998^56^ | 43 | 39 | NS | 20-24 | NS | Y | N | N | small VSD |
| 4. Qian Y, 2022 | 144 | NS | NS | 26.4* | NS | Y 144 | Y 7 | Y 11 | Other diseases |

**Supplementary Table 7. Summary of data for studies reporting about enlarged FGB.** PN: postnatally; GA: mean* gestational age or range as stated (weeks); TOP: termination of pregnancy; NS: not stated; n: number of patients; m: male; f: female; Y: yes; N: no; VSD-ventricular septal defect.
